# In Situ Monitoring of Morphology Changes and Oxygenation State of Human Erythrocytes During Surfactant-Induced Hemolysis

**DOI:** 10.3390/cells14070469

**Published:** 2025-03-21

**Authors:** Miroslav Karabaliev, Boyana Paarvanova, Gergana Savova, Bilyana Tacheva, Radostina Georgieva

**Affiliations:** 1 Department of Physics and Biophysics, Faculty of Medicine, Trakia University, 11 Armeiska, 6000 Stara Zagora, Bulgaria; boyana.parvanova@trakia-uni.bg (B.P.); gergana.savova@trakia-uni.bg (G.S.); bilyana.tacheva@trakia-uni.bg (B.T.); 2 Institute of Transfusion Medicine, Charité-Universitätsmedizin Berlin, Charitéplatz 1, 10117 Berlin, Germany

**Keywords:** erythrocytes, hemolysis, saponin, SDS, Triton X-100, hemoglobin, oxygenation

## Abstract

Erythrocytes, the most abundant blood cells, are a prevalent cell model for the analysis of the membrane-damaging effects of different molecules, including drugs. In response to stimuli, erythrocytes can change their morphology, e.g., shape or volume, which in turns influences their main function to transport oxygen. Membrane active molecules can induce hemolysis, i.e., release of hemoglobin into the blood plasma. Free hemoglobin in the blood circulation is toxic causing serious health problems including vasoconstriction, high blood pressure and kidney damage. Therefore, early recognition of the risk of massive hemolysis is highly important. Here, we investigated surfactant induced hemolysis applying UV–vis spectrophotometry. Saponin, sodium dodecyl sulfate and Triton X-100, detergents known to provoke hemolysis at different concentrations and by different mechanisms, were applied to initiate the process. Whole absorption spectra of erythrocyte suspensions in the range 300–750 nm were recorded every 15 s for following the process in real-time. The hemolysis process, with respect to morphological changes in the erythrocytes and their influence on the oxygenation state of hemoglobin, was characterized by the absorbance at 700 nm, the height relative to the background and the wavelength of the Soret peak. The results suggest that these UV–vis spectrophotometry parameters provide reliable information in real-time; not only about the process of hemolysis itself, but also about pre-hemolytic changes in the erythrocytes, even at sub-hemolytic surfactant concentrations.

## 1. Introduction

Erythrocytes, known as red blood cells (RBCs), are the most abundant blood cells mainly responsible for oxygen transport from the lungs to the tissues in the organism. Correspondingly, their main component is the iron-containing protein, hemoglobin, which has a high oxygen-binding capacity of 1.34 mL O_2_ per gram protein [1]. Additionally, the simple structure with no nuclei and other organelles, as well as the reduced cytoskeleton consisting of only an under-membrane protein network, allow an immense deformability that is necessary for passing, even through the smallest capillaries in the body. Erythrocytes are a unique and convenient biological model that is widely used in studies on the biophysical properties of biological membranes in general [2,3,4,5]. They allow for the in-depth exploration of the biophysical mechanisms of action of, for example, nanoparticles and the harmful effects of various molecules including drugs, as well as the investigation of membrane changes induced by them. Many authors also suggest that erythrocytes are highly promising as a natural drug carrier system [6,7,8].

Erythrocytes respond to such molecules, as well as to other environmental factors like ionic strength, osmotic pressure, pH, temperature, etc., mainly by changes in their shape, volume or area to volume ratio. Normally, in the blood plasma, they have a biconcave disk-like form (discocytes), but changing conditions can change them to spherical cells with an abnormal membrane (echinocytes), cup-shaped cells (stomatocytes), or to a spherical shape with an increased volume (spherocytes). The morphological response to different stimuli can be deformation or transformation. The process of deformation occurs with the senescence of the cells, but it can be reversible if it is induced [9]. Transformation, also known as poikilocytosis, is related to echinocytosis, stomatocytosis or spherocytosis, and can be reversible or not [10]. These shape changes are induced by various factors like pH, non-physiological salt concentrations, cholesterol enrichment or depletion, presence of cationic amphipaths etc., [11,12]. The surface area, measured for normal discocytes (139.4 μm^2^), increases in echinocytes (143.4 μm^2^), but decreases in stomatocytes (96.3 μm^2^). The increased area is due to the spicules formation, but the decrease in surface area is caused by loss of lipids through microvesiculation [13].

The shape and/or volume modifications in combination with other factors can seriously affect the membrane stability, which can lead to hemolysis, causing the release of hemoglobin (Hb) into the blood plasma. Free Hb in the blood circulation is toxic and can cause serious health problems, including vasoconstriction and kidney damage [14]. Therefore, it is important to recognize early the presence of free Hb in the circulation and to define the cause and extent of hemolysis. UV–vis spectrophotometry provides a variety of options for analysis of this process. The amount of hemoglobin released into the blood plasma, as well as its oxygenation state, can be analyzed by end-point assays at certain wavelengths. The main peak, the so-called Soret’s peak, occurs at 415 nm for oxygenated hemoglobin (oxy-Hb), at 430 nm for deoxygenated (deoxy-Hb) and at 405 nm for oxidized or methemoglobin (met-Hb). Additionally, oxy-Hb has two Q-bands at 542 nm and 578 nm, deoxy-Hb has one additional maximum at 560 nm and met-Hb has a peak at 630 nm, but no bands between 530 and 580 nm [15,16,17]. However, the measurements of erythrocyte suspensions in real-time are more informative because the induced morphological changes in the cells, together with the release of hemoglobin, affect the refractive index of the cells, and as a consequence, impact the light scattering of the erythrocyte suspension. In previous investigations, we analyzed the spectra of erythrocyte suspensions at different osmotic pressures regarding relative height and wavelength of the Soret’s peak, showing that these parameters are strongly impacted by the changes in the shape and volume of the cells [18]. More interestingly, the changes in light scattering measured in “real-time” provide information about shape or volume changes in the cells, especially in the pre-hemolytic state [19,20].

Detergents (surfactants) are often used to impact the stability of the cell membranes for different purposes, including disinfection, extraction of cytoplasmic compounds, cell membrane permeabilization, etc. They are amphiphilic molecules which can interact and disturb cell membranes by modifying the lipid and/or the protein part of the membrane. The intercalation with surfactants damages the erythrocyte membrane, which is accompanied by formation of echinocytes or stomatocytes [21]. The shape change of the erythrocytes is largely determined by the different charges of the applied surfactants. Anionic and neutral surfactants penetrate in the neutral external leaflets of the membrane, and are echinocytogenic. In contrast, cationic detergents are stomatocytogenic, because of the penetration into the inner part of the lipid bilayer, which is negatively charged due to the presence of phosphatidylserine [22]. The changes induced by different surfactants can range from small changes in membrane permeability to major effects such as cell lysis, depending on type, concentration and interaction time with the membrane [23]. Two main pathways of hemolysis by surfactants are suggested—a colloid–osmotic mechanism and solubilization—but the boundary between them still remains unclear. The colloid–osmotic mechanism is based on the induction of structural changes in the membrane, an increase in membrane permeability, colloid–osmotic swelling and cell rupture [22,24]. The process of solubilization is presented as a three-step model with initial penetration into the lipid bilayer, saturation with formation of mixed micelles of surfactant and lipids, and finally, the complete destruction of the bilayer structures [24,25,26,27].

The aim of the work presented here was to analyze the process of hemolysis initiated by three different surfactants, saponin, Triton X-100 and sodium dodecyl sulfate (SDS), which are described to cause different pre-hemolytic changes in erythrocytes and work by different mechanisms [24,28,29,30]. We followed the process from the initial interactions between detergents and erythrocytes in real-time using spectrophotometric analysis and focusing on the pre-hemolytic changes in the obtained spectra. Three spectral parameters are selected to estimate the pre-hemolytic changes in the morphology of RBCs—the absorbance at 700 nm, the Soret’s peak height relative to the spectrum background and the Soret’s peak wavelength.

## 2. Materials and Methods

### 2.1. Materials

Triton X-100 and Saponin weissrein were purchased from Merck, Darmstadt, Germany; PBS—phosphate-buffered saline (tablets, pH 7.4 at 25 °C), SDS—sodium dodecyl sulfate and NaCl (sodium chloride) from Sigma-Aldrich, St. Louis, MO, USA.

### 2.2. Isolation of Erythrocytes

The erythrocytes were isolated from fresh blood samples of healthy donors and taken into vials with EDTA according to the protocol No. 30/25 April 2024 of the ethics commission of the Medical Faculty, Trakia University, Stara Zagora, Bulgaria. Informed consent was obtained from all subjects donating blood for the study. After centrifugation at 1700× *g* for 3 min, the blood plasma was removed, together with the layer of white blood cells on top of the erythrocyte sediment. Isolated erythrocytes were washed twice in isotonic 150 mM NaCl saline, and finally resuspended in the washing saline at a hematocrit (Hct) of 7.5% or 10%.

### 2.3. Measurements of Hemolysis in Real-Time

Initial detergent solutions were prepared in saline to final concentrations of 1% *v*/*v* for Triton X100; 1% *w*/*v* for saponin and 0.1% *w*/*v* for SDS. Just before each experiment, 3 mL of the detergent solution in PBS with the required concentration was prepared in the quartz cuvette, with an optical path length of 1 cm. For the experiments with saponin and Triton X-100, 20 µL of RBCs suspension with a Hct 7.5% were added to obtain suspensions with a final Hct of 0.05%. For the experiments with SDS 20 µL of RBCs suspension with Hct, 10% were added, and the final concentration during measurements was 0.07%. The suspension was homogenized immediately, and the cuvette was placed in a Cary 60 UV–vis spectrophotometer (Agilent Technologies, Santa Clara, CA, USA) to measure the light absorption. At the end of the process, the RBCs were hemolyzed with a high concentration of the detergent and the spectrum of the released hemoglobin was measured. The samples were scanned every 15 s in the wavelength range from 300 nm to 750 nm at an interval of 0.5 nm. To determinate the pre-hemolytic changes, we used several parameters obtained from the absorption spectra, as described in our previous work [18].

#### 2.3.1. Absorbance at 700 nm (A700)

At this wavelength, there is no specific absorption by hemoglobin or other erythrocyte components. The result of measured absorption is due to light scattering by intact RBCs. Scattering reduces the intensity of the light passed through the sample and leads to higher absorbance values without real absorption by the erythrocytes. Pre-hemolytic changes in RBCs volume or shape caused by the detergent lead to changes in the intensity of scattered light and hence to changes at the measured A700.

#### 2.3.2. Soret’s Peak Height

The Soret’s peak is due to absorption of light by hemoglobin. Peak height is directly proportional to hemoglobin concentration. In intact erythrocytes, hemoglobin can absorb only the light that penetrates into the cell. Due to the high intensity of light scattered by erythrocytes in suspension, the height of the Soret’s peak is related to both the absorption of hemoglobin and the background of the spectrum due to scattering. In order to ignore light scattering, we used the relative height of the Soret’s peak, which is obtained as the difference between the measured absorbance at the peak wavelength and the measured absorbance at a wavelength of 500 nm where hemoglobin does not absorb.

#### 2.3.3. Soret’s Peak Wavelength

The applied scan step of 0.5 nm does not allow the correct determination of the wavelength of the Soret’s peak. For this reason, we used the first derivative of the absorption spectrum (∂A/∂t). The procedure was described in details previously [31]. The wavelength at which the first derivative has a zero value in the range of the Soret’s peak corresponds to the exact wavelength of the peak.

### 2.4. Light Microscopy (LM)

RBCs were imaged using an inverted fluorescence microscope (Olympus CKX41 with Olympus SC50 camera, Olympys Corporation, Tokyo, Japan). A drop of each RBCs suspension was taken directly from the cuvette and imaged in parallel with the spectrophotometric measurement.

## 3. Results and Discussion

The action of the detergents, saponin, SDS and Triton X-100 on erythrocytes was studied by microscopy and UV–vis spectroscopy. These detergents are different by electric charge and cause hemolysis at different concentrations. It is known that before hemolysis they penetrate in the lipid bilayer of the membrane and can cause changes in the shape of the erythrocytes.

### 3.1. Saponin

Saponins are used in folk medicine from ancient times because of their anti-inflammatory [32], antiviral [33,34], antifungal [35], antimicrobial [36] and antitumor [37] properties. Saponins are organic chemicals composed of triterpene or steroid aglycones and sugar side chains, and because of their amphiphilic structure, they are well-known lytic agents against RBCs. They are able to form complexes with cholesterol, which can create specific pores in the membrane [38] and subsequent cell lysis, most likely by a colloid–osmotic mechanism [39].

**Figure 1:**
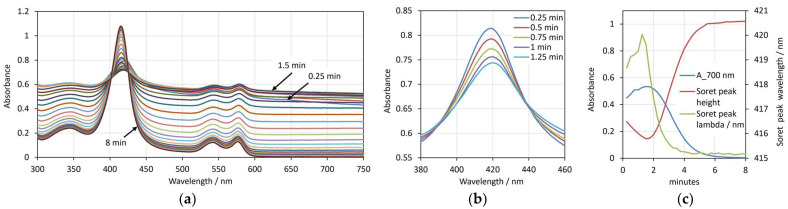
(**a**) Spectra of erythrocyte suspension with 0.05% Hct in the presence of 20 ppm saponin during the overall process of hemolysis for up to 8 min. (**b**) Spectra in the range 380–460 nm taken at 0.25 min, 0.5 min, 1 min, 1.25 min, 1.5 min after addition of saponin. (**c**) Changes in the absorbance at 700 nm, the Soret peak relative height and the Soret peak wavelength during the overall 8 min process of hemolysis. Spectra are measured at time interval 15 s.

The entire absorption spectra, taken each 15 s during a process of hemolysis caused by saponin, are shown in Figure 1a. With intact erythrocytes, the scattering is at its highest value, and the light transmission through the sample is at the lowest. Correspondingly, before the beginning of hemolysis, the absorption spectrum has a relatively high background at all wavelengths. Due to the forward scattering, a portion of the light does not penetrate the erythrocytes, and is not absorbed by the hemoglobin inside. During hemolysis, due to the decreasing number of intact erythrocytes, the light scattering decreases, and the background also decreases. At the same time, the amount of hemoglobin released into the suspension medium increases, which results in higher Soret and Q-band absorption peaks.

Figure 1b shows excerpts of the spectra (in the range of the Soret peak) taken during the initial stage of the process up to 1.25 min. It can be seen that during this period, the changes in the spectra are in the opposite direction compared to the main stage of the hemolysis process. Namely, the background due to the light scattering increases and the Soret peak height decreases in its absolute value, as well as when expressed as height relative to the background of the spectrum. A slight shift in the peak wavelength can also be noticed.

For comparison, the changes in the three spectrum parameters during the overall process of the hemolysis caused by saponin are shown in Figure 1c. The process of real hemolysis, which starts after approximately 1.5 min, is clearly expressed by the decrease in the absorbance at 700 nm (the blue curve) and the increase in the Soret peak relative height (the red curve). Since the pre-hemolytic changes in the spectra are in directions opposite to the hemolytic changes, all tree curves express an extremum, i.e., the background and the Soret peak wavelength have maxima, while the Soret peak relative height has a minimum.

From these results, we can make the suggestion that in the pre-hemolytic phase, saponin causes changes in the erythrocyte shape that are consistent with a decreased volume. The relation of the three discussed spectral parameters with the decreased volume of the erythrocytes was demonstrated in our previous work, where the volume of the erythrocytes was decreased by high osmotic pressure [18].

**Figure 2:**
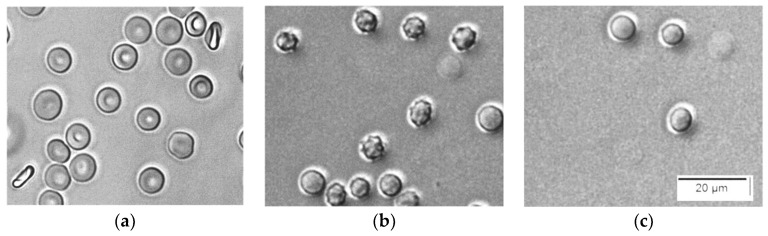
Consecutive microscopic images of the process of hemolysis caused by saponin. (**a**) Suspension of erythrocytes in PBS, Hct 0,05%. (**b**) 1 min after the addition of 25 ppm saponin; (**c**) 5 min after the addition of 25 ppm saponin.

This is in agreement with the microscopic images taken during a process of hemolysis caused by saponin (Figure 2). It is seen that initially, erythrocytes in PBS are discocytes (Figure 2a), but within 1 min after the addition of saponin, they are turned to echinocytes (Figure 2b). Thereafter, the echinocytes inflate to become spherocytes and start to hemolyze (Figure 2c). The apparent inflation indicates that the colloid–osmotic mechanism is responsible for the hemolysis.

**Figure 3:**
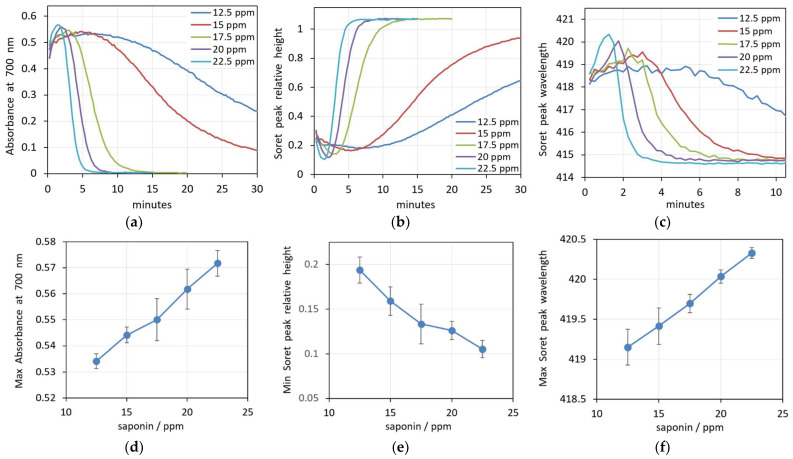
(**a**–**c**) Changes in spectra parameters during hemolysis caused by five increasing concentrations of saponin in 0.05% Hct suspensions. (**a**) Absorbance at 700 nm, (**b**) Soret peak height, (**c**) Soret peak wavelength. (**d**–**f**) Concentration dependence of the prehemolytic values of spectra parameters. (**d**) Maximum absorbance at 700 nm, (**e**) Soret peak height at its minimum, (**f**) Soret peak wavelength at its maximum. (n = 5).

In addition, we also analyzed these processes at different saponin concentrations (Figure 3a–c). It can be seen that for all hemolytic concentrations of saponin, the process runs in a similar manner and the parameters undergo similar changes. For all concentrations, there is a pre-hemolytic change expressed by an increase in the spectrum background, a decrease in the Soret peak height and an increase in the Soret peak wavelength.

The initial values of all three parameters are very close for the different saponin concentrations, suggesting relatively slow initial processes. The rate and degree of the changes that precede the hemolysis are obviously dependent on the saponin concentration, with the bigger concentration causing faster and more prominent changes. The graphs in Figure 3d–f summarize the extent of prehemolytic changes in the parameters at the applied concentrations of saponin. It is obvious that at the given experimental conditions, there are linear concentration dependencies for all three spectra parameters.

### 3.2. SDS

**Figure 4:**
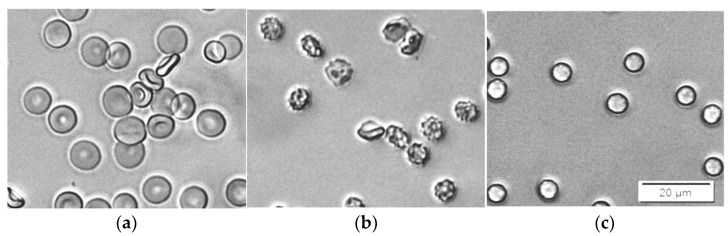
Microscopic images demonstrate the effect of SDS on the shape of erythrocytes. The suspensions contain erythrocytes in PBS at Hct 0.07% and: (**a**) 0 ppm SDS; (**b**) 0.33 ppm SDS; (**c**) 3.33 ppm SDS.

Sodium dodecyl sulfate (SDS) is an anionic detergent familiar with antiviral and antibacterial properties [40]. Its incorporation in the erythrocyte membrane also results in shape change from discocyte to an echinocyte type at low concentrations. At higher concentrations, erythrocytes are turned to spherocytes (Figure 4). For a period of 30 min, no hemolysis was observed for concentrations up to approximately 1.5 ppm SDS in erythrocyte suspensions with 0.07% Hct.

**Figure 5:**
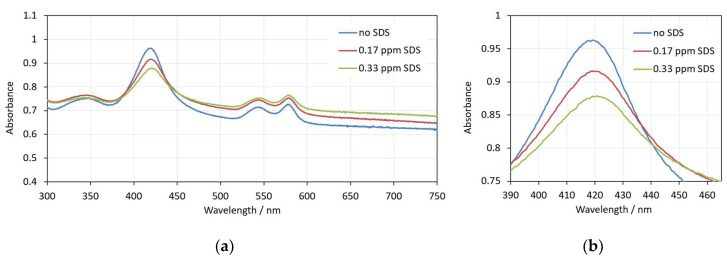
(**a**) Spectra of erythrocyte suspension with 0.07% Hct in the presence of different amounts of SDS—no SDS, 0.17 ppm SDS and 0.33 ppm SDS; (**b**) enlarged view of the same spectra in the range 390–460 nm. The spectra are measured at the 30th minute after addition of SDS to the erythrocytes.

Figure 5 shows the spectra of erythrocytes suspensions taken at the 30th minute after addition of SDS at low, pre-hemolytic concentrations. As it can be seen, the interaction with SDS affects the appearance of the spectra. The background increases, the height of the Soret peak decreases, and the peak wavelength shifts to larger values. These changes correspond to increased light scattering, reflecting the shape and volume transformations of the erythrocytes.

**Figure 6:**
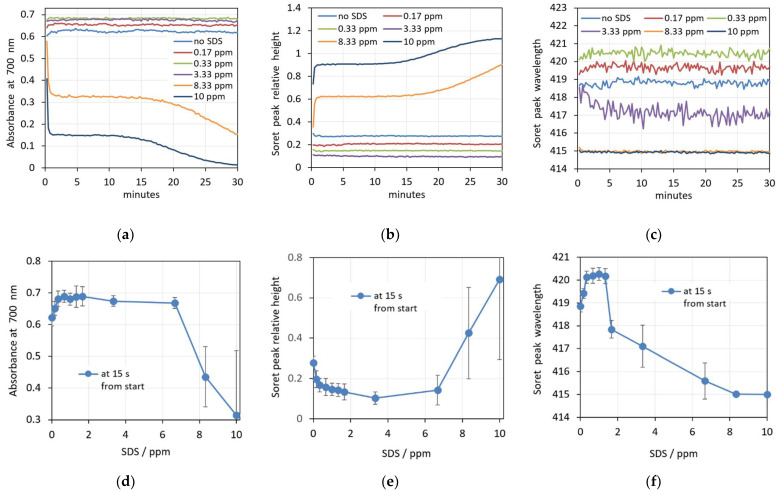
(**a**–**c**) Changes in the spectral parameters after the addition of five increasing concentrations of SDS: (**a**) absorbance at 700 nm; (**b**) Soret peak relative height; (**c**) Soret peak wavelength. (**d**–**f**) Concentration dependence of the prehemolytic values of spectra parameters taken 15 s after the addition of SDS: (**d**) maximum absorbance at 700 nm, (**e**) Soret peak height at its minimum, (**f**) Soret peak wavelength at its maximum. (n = 5).

The real-time changes in the three spectral parameters caused by five increasing concentrations of SDS are shown in Figure 6a–c. Contrary to the case with saponin, here the changes in the spectral parameters are very fast, and at the beginning of the measurements, they are already different for different SDS concentrations. At the three lowest concentrations (0.17 ppm and 0.33 ppm and 3.33 ppm), the scattering during the whole period of 30 min measurement remains constant, which is reflected in the graphs in Figure 6, where the red, green and violet curves are parallel to the control without SDS (blue curve). With an increasing concentration, there is a slightly increased background and decreased relative height of the Soret peak. This behavior corresponds to the observed shape and volume changes mentioned above.

The Soret peak wavelength is also shifted to higher values for the SDS concentrations of 0.17 and 0.33 ppm, but drops by approximately 3 nm at an SDS concentration of 3.33 ppm. This result is quite interesting because it clearly indicates the initiation of more dramatic changes in the cells at this concentration before the start of the real hemolysis.

A further increase in the SDS concentration causes the acceleration of the damaging processes, and the change in the spectra parameters goes into the opposite direction (orange and dark blue curves in Figure 6a–c), reflecting the start of real hemolysis, with the release of hemoglobin into the suspension medium. However, here the process seems to be more complicated, including two periods with different kinetics of the hemolysis. The background absorbance at 700 nm starts at some initial values and decreases very fast during the first 45 s (Figure 6a). The same can be noticed in the curves for these concentrations in Figure 6b, where the Soret peak height increases quickly during this period. The Soret peak wavelength also drops very quickly, and stays at 415 nm, indicating an increase in the oxygenation of Hb, which is typically caused by its release out of the erythrocytes. This initial changes are obviously due to some initial partial hemolysis. Thereafter, a lag period of up to 15 min is observed with constant spectral parameters, followed by the final process up to the entire hemolysis of the erythrocytes.

The results suggest a more complex process of interaction of SDS with the erythrocyte membrane. SDS is assigned as a slow solubilizing agent [29]. The anionic SDS needs longer time (minutes or hours) to penetrate into the membrane due to its repulsion by the negatively charged glycocalyx [41]. Shalel et al. found that as the ionic strength increases, the SDS adsorption to the membrane also increases. This increases the amount of membrane-bound surfactant to such an extent that it results in solubilization rather than the simple enhancement of the membrane permeability [24].

Bielawski suggested that, at certain concentrations of SDS, hemolysis is a two-level process [42]. First, a fraction of the erythrocytes is quickly hemolyzed by the so-called damage effect. There is a membrane disruption and increased permeability caused by changes in the organization of the membrane components with the detergent. In a single erythrocyte, this change is an all-or-none process. After this period of time, the remaining erythrocytes are hemolyzed by the colloid–osmotic mechanism. The duration of this period, as well as the amount of the hemolyzed fraction during the first period, depend on the SDS concentration. This hypothesis is in good agreement with our results. The images in Figure 4b,c show that during this process, the erythrocytes that remain intact are all already spherocytes, and remain intact and stable for a certain period of time.

The concentration dependence of the prehemolytic values of spectra parameters after the addition of SDS are shown in Figure 6d–f. Several concentration regions can be distinguished from these graphs. In the lowest concentration region, from 0 to 0.33 ppm SDS concentration, the spectra parameters change linearly, reflecting the change in shape and volume of erythrocytes. A further increase in SDS concentration up to 1.33 ppm does not seem to cause more changes in the erythrocytes’ shape and volume according to the constant values of all three spectra parameters. Above 1.33 ppm, a significant decrease in the Soret peak wavelength is observed (Figure 6f). This could be explained by the start of Hb release in small amounts from the cells during their rapid transition from echinocytes to spherocytes. The free Hb in the suspension medium is more oxygenated, causing the shift in the Soret peak wavelength. It is interesting to note that this process is not revealed by the other two spectra parameters, the absorbance at 700 nm and the Soret peak relative height. At SDS concentrations above 6.66 ppm, the fractional hemolysis discussed above is reflected by all three spectra parameters.

It should be emphasized that all values in Figure 6d–f are measured at as short a time as possible after the addition of SDS, reflecting the rapid initial changes in the erythrocytes shape and volume upon the action of SDS.

### 3.3. Triton X-100

Triton X-100 is a non-ionic surfactant that, similarly to saponins, shows mainly antiviral [43,44] and antitumor [45,46] activities. It has been shown that membrane domains that are rich in cholesterol or similar molecules are more tolerant to Triton X-100 than other parts of the lipid bilayer [47]. At low concentrations, Triton X-100 has been reported to stabilize erythrocytes under hypotonic conditions, but it becomes a lytic agent at higher concentrations [30].

**Figure 7:**
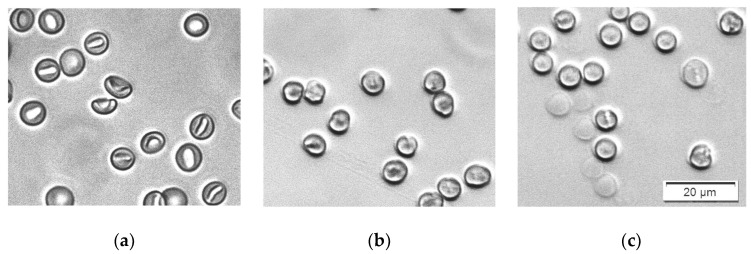
Microscopic images of the process of hemolysis caused by Triton X-100. The suspension contains erythrocytes in PBS to Hct 0,05%, and (**a**) 10 ppm Triton X-100; (**b**) 75 ppm Triton X-100 at 0 min after addition; and (**c**) 75 ppm Triton X-100 at 5 min after addition.

Differently from the previously described effects of saponin and SDS, we detected changes in the shape of erythrocytes from discocyte to stomatocyte at a concentration of 10 ppm Triton X-100 (Figure 7a). At higher concentrations of Triton X-100 (here 75 ppm), the stomatocytes transform to sphero-stomatocytes (Figure 7b), and approximately 5 min later (at the same Triton X-100 concentration), the erythrocytes swell and lyse (Figure 7c).

**Figure 8:**
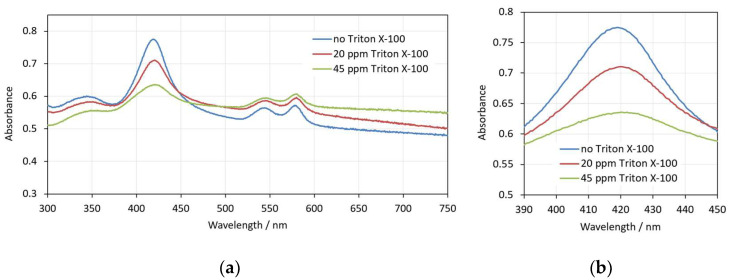
(**a**) Spectra of 0.05% Hct erythrocyte suspension in the presence of different amounts of Triton X-100—no Triton X-100, 20 ppm Triton X-100 and 45 ppm Triton X-100; (**b**) enlarged view of the same spectra in the range 390–450 nm. The spectra are measured in the 30th minute after the interaction of the erythrocytes with Triton X-100.

As in the case of SDS, with pre-hemolytic concentrations of Triton X-100 (up to 50 ppm), there are changes in the shape and volume of erythrocytes that do not cause hemolysis for a relatively long period of time (here detected for at least 30 min). In Figure 8, three spectra that reflect these changes for concentrations of up to 45 ppm Triton X-100 are presented. Although the shape changes in the erythrocytes are different compared to SDS, the observed changes in the spectra are similar—the background increases, the Soret peak height decreases and the Soret peak wavelength increases with the increase in the detergent concentration.

**Figure 9:**
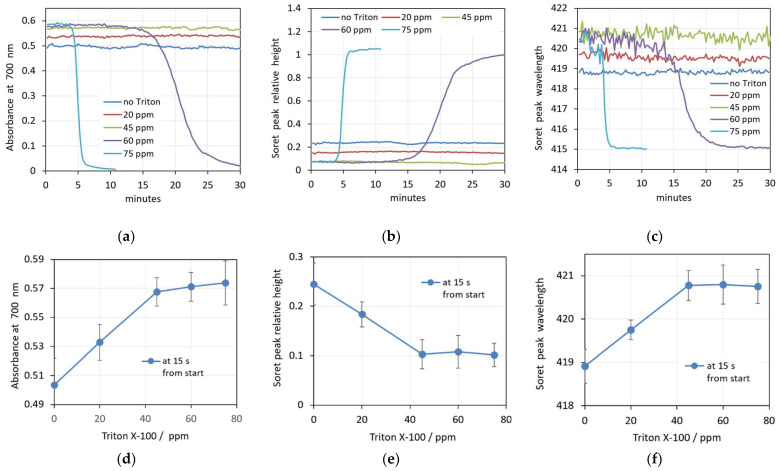
(**a**–**c**) Changes in the spectra parameters caused by increasing concentrations of Triton X-100 in 0.05% Hct suspensions. (**a**) Absorbance at 700 nm, (**b**) Soret peak height, (**c**) Soret peak wavelength. (**d**–**f**) Concentration dependence of the prehemolytic values of spectra parameters taken 15 s after addition of Triton X-100. (**d**) Maximum absorbance at 700 nm, (**e**) Soret peak height at its minimum, (**f**) Soret peak wavelength at its maximum. (n = 5).

The real-time changes in the spectral parameters caused by four increasing concentrations of Triton X-100 are shown in Figure 9a–c. Similarly to the SDS action, the changes in the erythrocyte shape and volume upon interaction with Triton X-100 at the lower concentrations (20 ppm and 45 ppm) are very fast, as reflected in the constant scattering during the whole 30 min period of measurement. The green and red curves are parallel to the control without Triton X-100 (blue curve) in the graphs shown in Figure 9a–c. With increasing concentrations, there is a slightly increased background (absorbance at 700 nm) and decreased relative height of the Soret peak. The wavelength of the Soret peak increases, indicating lower oxygenation of the Hb during shrinking of the cells. This behavior corresponds to the observed shape and volume changes mentioned above.

The concentration dependence of the prehemolytic values of spectra parameters after the addition of Triton X-100 are shown in Figure 9d–f. It can be seen that up to a concentration of 45 ppm Triton X-100, the observed values of all three parameters change linearly, while above 45 ppm and up to 75 ppm, the parameters remain constant. This can also be seen in Figure 9a–c, where the curves for concentrations above 45 ppm Triton X-100 start at very close values. However, while 45 ppm Triton X-100 keeps the spectra parameters constant for 30 min, concentrations of 60 ppm and 75 ppm Triton X-100 cause hemolysis. This is detected as a quickly decreasing background (decreasing absorbance at 700 nm with simultaneously increasing relative height of the Soret peak) and a drop of the Soret peak wavelength from 421 to 415 nm, indicating changes in the oxygenation state of the released Hb.

Usually, Triton X-100 is considered as a fast-solubilizing detergent, which can flip from the outer to the inner layer of the membrane and can equilibrate in both layers within milliseconds or seconds, causing a rapid solubilization [28,29,41]. Our results show that Triton X-100 is really a fast-acting detergent over the whole range of concentrations used in the work (20 ppm to 75 ppm in relation to erythrocyte suspensions with 0.05% Hct). All used concentrations induce fast spectral changes, as demonstrated in Figure 9a–c. At the same time, the process of the real hemolysis occurring above a certain critical concentrations of Triton-X in the erythrocyte membrane starts after a certain lag period of time. The duration of the lag period decreases with increasing concentration, being approximately 15 min for 60 ppm Triton X-100 and only 4 min for 75 ppm Triton X-100. This can be explained by the longer equilibration time of the surfactant in the membrane for the lower concentration. Also, the kinetics of hemolysis depend on the detergent concentration. At 60 ppm, it takes more than 15 min to complete the hemolysis. In contrast, at 75 ppm, the whole process takes less than 3 min. Higher concentration of Triton X-100 in the membrane obviously causes formation of larger pores, which accelerates the release of hemoglobin from the erythrocytes. In contrast to the two-level process initiated by SDS above certain critical concentrations, here the hemolysis is a single-level process by the colloid–osmotic mechanism.

In general, when analyzing the kinetics of hemolysis, one has to consider that the population of erythrocytes in each individual healthy donor is inhomogeneous in terms of age, size, shape and oxygenation state, and contains a small proportion of immature red cells, reticulocytes. It is well established that older cells are the first to undergo hemolysis. With the senescence of the cells, they undergo several changes; for instance, their density increases [48] but their size decreases due to the loss of hemoglobin [49]. Furthermore, with advancing age, erythrocytes exhibit reduced deformability, increased cytosolic viscosity, and delayed shape recovery. Alterations in redox balance occur, accompanied by a decrease in the activity of certain enzymes [49,50,51,52,53]. Also, membrane asymmetry is disrupted by the translocation of phosphatidylserine (PS) from the inner to the outer layer of the cell membrane. Under normal conditions, PS is predominantly localized on the inner side; however, as erythrocytes age, the structural integrity of the cell membrane deteriorates, leading to the exposure of PS on the outer surface, which is a marker of the senescence of the cell [54,55,56,57]. On the other hand, the presence of PS (an anionic phospholipid) on the erythrocyte surface in higher amounts could change the electrostatic interaction with the anionic SDS, which in turn influences the hemolysis kinetics, eventually contributing to the biphasic character of the hemolysis process in this case.

Finally, it must be taken into account that there is a large inter-individual variation in the erythrocyte population, which originates from genetic and epigenetic factors. The differences include morphological variances, density, deformability, membrane stability, enzyme activity, etc. [58,59]. The inter-individual differences in membrane stability strongly influence the process of hemolysis. This is reflected by the standard deviations in Figure 3e–f, Figure 6e–f and Figure 9e–f, where we display the values of the investigated spectral parameters measured for the erythrocytes of five randomly selected healthy donors at increasing concentrations of saponin, SDS and Triton X-100, respectively. Especially at high SDS concentrations, the standard deviation of the parameters characterizing the scattering of the erythrocyte suspensions, the maximum absorbance at 700 nm and the minimum relative height of the Soret peak are extraordinarily high.

## 4. Conclusions

The results presented in this work demonstrate that the three detergents used provoke changes in the shape, volume and intactness of erythrocytes. These changes can be observed by microscopy, but can also be detected by measuring the UV–vis absorption spectra of the whole erythrocyte suspension during the overall process of hemolysis. The three detergents—saponin, SDS and Triton X-100—were selected for this investigation as they induce hemolysis by different processes and cause different pre-hemolytic changes in the cells. As our results suggest, the proposed method of continuous measurement of the absorption spectrum is suitable to monitor all stages of the pre-hemolytic and hemolytic processes with the real-time changes in the spectral parameters.

Erythrocytes, with their well-investigated membranes, provide the ideal model for these purposes. Especially in view of their promising potential application as drug delivery systems or as carriers for nanoparticles in diagnostics, the stability of the carrier erythrocytes and prediction of their circulation time are of great importance. We believe that, with our contribution, we suggest a valuable, inexpensive and easily available approach to study the effects, mechanisms and kinetics of the interaction of almost every compound of interest, including drugs, nanoparticles, etc., with biological membranes.

## Data Availability

The data are available upon request to the authors.
